# Red seaweed *(Asparagopsis taxiformis)* supplementation reduces enteric methane by over 80 percent in beef steers

**DOI:** 10.1371/journal.pone.0247820

**Published:** 2021-03-17

**Authors:** Breanna M. Roque, Marielena Venegas, Robert D. Kinley, Rocky de Nys, Toni L. Duarte, Xiang Yang, Ermias Kebreab

**Affiliations:** 1 Department of Animal Science, University of California, Davis, California, United States of America; 2 Commonwealth Scientific and Industrial Research Organisation, Agriculture and Food, Townsville, Queensland, Australia; 3 College of Science and Engineering, James Cook University, Townsville, Queensland, Australia; United States Department of Agriculture, Agricultural Research Service, UNITED STATES

## Abstract

The red macroalgae (seaweed) *Asparagopsis* spp. has shown to reduce ruminant enteric methane (CH_4_) production up to 99% *in vitro*. The objective of this study was to determine the effect of *Asparagopsis taxiformis* on CH_4_ production (g/day per animal), yield (g CH_4_/kg dry matter intake (DMI)), and intensity (g CH_4_/kg ADG); average daily gain (ADG; kg gain/day), feed conversion efficiency (FCE; kg ADG/kg DMI), and carcass and meat quality in growing beef steers. Twenty-one Angus-Hereford beef steers were randomly allocated to one of three treatment groups: 0% (Control), 0.25% (Low), and 0.5% (High) *A*. *taxiformis* inclusion based on organic matter intake. Steers were fed 3 diets: high, medium, and low forage total mixed ration (TMR) representing life-stage diets of growing beef steers. The Low and High treatments over 147 days reduced enteric CH_4_ yield 45 and 68%, respectively. However, there was an interaction between TMR type and the magnitude of CH_4_ yield reduction. Supplementing low forage TMR reduced CH_4_ yield 69.8% (*P* <0.01) for Low and 80% (*P* <0.01) for High treatments. Hydrogen (H_2_) yield (g H_2_/DMI) increased (*P* <0.01) 336 and 590% compared to Control for the Low and High treatments, respectively. Carbon dioxide (CO_2_) yield (g CO_2_/DMI) increased 13.7% between Control and High treatments (P = 0.03). No differences were found in ADG, carcass quality, strip loin proximate analysis and shear force, or consumer taste preferences. DMI tended to decrease 8% (*P* = 0.08) in the Low treatment and DMI decreased 14% (*P* <0.01) in the High treatment. Conversely, FCE tended to increase 7% in Low (*P* = 0.06) and increased 14% in High (*P* <0.01) treatment compared to Control. The persistent reduction of CH_4_ by *A*. *taxiformis* supplementation suggests that this is a viable feed additive to significantly decrease the carbon footprint of ruminant livestock and potentially increase production efficiency.

## Introduction

Livestock production, particularly ruminants, contributes to anthropogenic greenhouse gas (GHG) emissions globally. These emissions are estimated to be 7.1 Gt carbon dioxide (CO_2_) equivalents annually which accounts for approximately 14.5% of the global anthropogenic GHG emissions [1]. The majority of GHG emissions from livestock production is in the form of methane (CH_4_), which is produced largely through enteric fermentation and to a lesser extent manure decomposition. Enteric CH_4_ emissions not only contribute to total agricultural GHG emissions but also represent an energy loss amounting up to 11% of dietary energy consumption [2]. Therefore, reducing enteric CH_4_ emissions decreases the total agricultural contribution to climate change and can improve productivity through conservation of feed energy. There is potential for mitigation of enteric CH_4_ emissions through a variety of approaches with a focus on the use of feed additives, dietary manipulation and forage quality [3].

Feed additives used in CH_4_ mitigation can either modify the rumen environment or directly inhibit methanogenesis resulting in lower enteric CH_4_ production (g/day per animal) and yield (g/kg dry matter intake [DMI]). Reductions in CH_4_ production of beef cattle, through the direct inhibition of methanogenesis, have been reported for feed additives at 22, 93, and 98% for short-chain nitro-compounds (3-nitrooxypropanol; 3-NOP, [4]), synthetic halogenated compounds [5], and naturally synthesized halogenated compounds in seaweed [6], respectively. The compound 3-NOP inhibits the enzyme methyl-coenzyme M reductase (MCR) which catalyzes the final step in methanogenesis in rumen archaea [7]. Halogenated CH_4_ analogs, such as bromoform, act on the same methanogenesis pathway by binding and sequestering the prosthetic group required by MCR in order to form CH_4_ [8–10]. Some haloalkanes are structural analogs of CH_4_, and therefore competitively inhibit the methyl transfer reactions that are necessary in CH_4_ biosynthesis [11,12]. These CH_4_ analogues include bromochloromethane (BCM), bromoform, and chloroform and have been proven to be most effective for reducing CH_4_ production. A 93% reduction of CH_4_ was shown in Brahman cattle with a feed inclusion of BCM at 0.30 g/100 kg LW twice daily for 28 days, however feed intake, weight gain, carcass quality or feed efficiency were not statistically different [5]. Conversely, Abecia et al. (2012) reported that the inclusion of BCM at 0.30 g/100 kg once per day decreased CH_4_ production 33% and increased milk production 36% [13]. The authors speculated that increased milk production in BCM treated cows could be attributed to a shift to more propionate production in the rumen, which is a hydrogen (H_2_) sink and provides more energy compared to other volatile fatty acids. However, long-term efficacy of CH_4_ analogues in the rumen remains to be confirmed. For example, Tomkins et al. (2009) reported a second experiment resulting in a 57.6% CH_4_ reduction after 30 days of treatment which is far less than the reductions found during the first 28 days [5]. Additionally, chloroform fed to fistulated dairy cows was effective at reducing enteric CH_4_ production through reduced abundance and activity of methanogenic archaea, but only over a 42-day period [14].

Types of feedstuffs can also impact CH_4_ production by providing different substrates to microbial populations which are the drivers of volatile fatty acid (VFA) production in the rumen. There are ways to influence the types of VFA produced in the rumen by changing the types of feed in the diet [15,16]. This is important for two reasons; first VFAs are utilized as an energy source for animal productivity and second VFA pathways, such as the production of propionate, are able to utilize reducing equivalents that normally would be shifted to methanogenesis [17,18]. Concentrates contain non-structural carbohydrates, such as starch and sugar, that are rapidly fermented which drives pH down, negatively impacting methanogenic populations, and are an effective way to increase propionate production [19,20]. Forages contain structural carbohydrates, such as neutral detergent fiber (NDF), and have been linked to increased CH_4_ production [21]. As dietary NDF increases, rumen pH also increases resulting in preferential production of acetate over propionate, which generates reducing equivalents that are then used in the methanogenesis pathway [22,23]. Fiber content in feeds play a significant role in CH_4_ production, including impacting the efficacy of anti-methanogenic compounds, such as 3-NOP and bromoform that specifically target MCR [4]. This hypothesis is based on the assumption that when high grain diets are fed, NDF decreases and ruminal MCR concentration is likely lowered thus granting greater efficacy for anti-methanogenic compounds to target a greater proportion of MCR which results in greater methane reductions [24].

Some red seaweeds are anti-methanogenic, particularly the genus *Asparagopsis*, due to their capacity to synthesize and encapsulate halogenated CH_4_ analogues, such as bromoform and dibromochloromethane, within specialized gland cells as a natural defense mechanism [25]. In a screening process to identify CH_4_ reduction potential of select macroalgae in Australia, *Asparagopsis taxiformis* was demonstrated to be the most promising species with a 98.9% reduction of CH_4_ when applied at 17% OM *in vitro* [26]. Although that level of inclusion of seaweeds is not practical for livestock production, subsequent studies demonstrated effective inclusion levels below 2.0% OM for *Asparagopsis in vitro* [27,28] without affecting total VFA concentrations or substrate digestibility. There are only two published studies that measured CH_4_ reduction by supplementing *Asparagopsis* in cattle diets. Reductions in CH_4_ as high as 98% were reported when *A*. *taxiformis* (containing 6.6 mg bromoform/g DMI) was supplemented at 0.2% OM in a high concentrate feedlot TMR [6]. In dairy, a 67% CH_4_ reduction was observed when *Asparagopsis armata* (at 1.3 mg bromoform/g DMI) was supplemented at 1% OM over a two-week feeding period [29]. The differences in efficacy between the two studies were the concentration of bromoform in the naturally variable wild harvested seaweed and diet formulation (high grain versus low grain) [6]. *A*. *taxiformis* reduces CH_4_ more effectively compared to similar inclusions of pure bromoform *in vitro* probably be due to multiple anti-methanogenic CH_4_ analogues working synergistically in the macroalgae [30]. Furthermore, *A*. *taxiformis* synthesizes multiple anti-methanogenic CH_4_ analogues such as bromo- and iodo- methanes and ethanes [31] and that methanogen species are differentially sensitive to CH_4_ inhibitors [32].

For adoption of the seaweed by industry it is crucial that meat quality be maintained or improved. As with any feed additive, feeding *A*. *taxiformis* to livestock has the potential to alter meat quality, tenderness, taste, and consumer acceptability. Marbling, for instance, directly impacts flavor and juiciness and it has been shown that marbling can directly influence consumer preference with some willing to pay a premium [33].

We hypothesize that a significant anti-methanogenic effect of *A*. *taxiformis* would 1.) persist throughout introduction, transition, and finishing periods in a typical beef feedlot scenario, 2.) have no detrimental effects on animal productivity or meat quality and 3.) not contain bromoform residues within the meat and liver would be present.

## Materials and methods

This study was approved by the Institutional Animal Care and Use Committee of the University of California, Davis (Protocol No. 20803).

### Study design, animals, and diets

Twenty-one Angus-Hereford cross beef steers, blocked by weight, were randomly allocated to one of three treatment groups: 0% (Control, n = 7), 0.25% (Low, n = 7), and 0.5% (High, n = 6) inclusion rates of *A*. *taxiformis* based on OM intake. The unbalanced number of steers between treatment groups was due to an unexpected animal injury during the last three weeks of the trial to which all data from this steer was removed from statistical analysis. The steers used in this study were obtained from the Shasta Livestock Auction Yard (Cottonwood, CA), all of which were sourced from the same ranch, and were approximately 8 months of age weighing approximately 352 ± 9 kg at the start of the trial. Each steer was randomly assigned to an individual pen, fitted with its own feed bunk, and were fed twice per day at 0600 and 1800 hours at 105% of the previous day’s intake.

**Fig 1:**
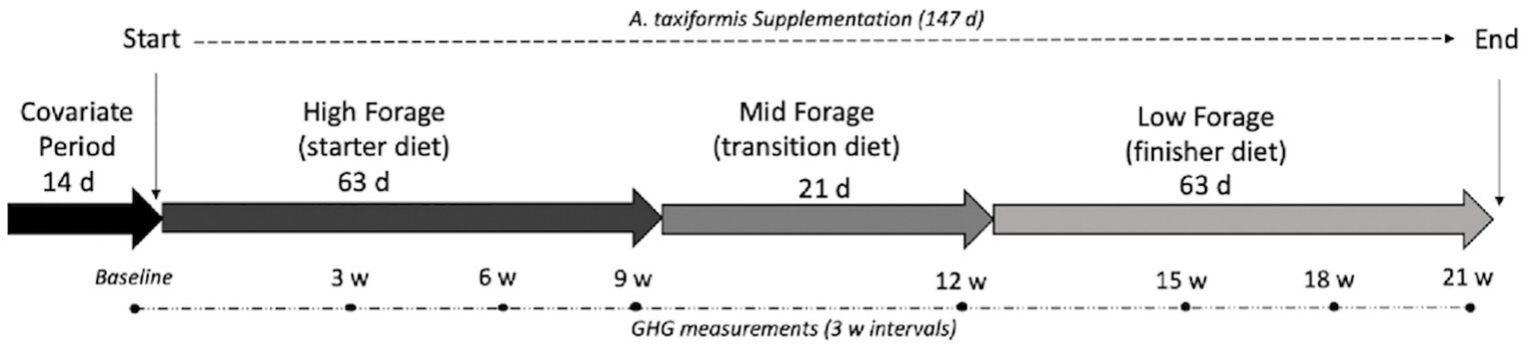
Experimental timeline including covariate period, *Asparagopsis taxiformis* implementation, dietary regime, and greenhouse gas measurement intervals.

The experiment followed a completely randomized design, with a 2-week covariate period, used as a baseline period, before treatment began followed by 3-week data collection intervals for 21-weeks; a total of 147 days (Fig 1). During data collection intervals, alfalfa pellets offered through the gas measuring device (GreenFeed system, C-Lock, Inc., Rapid City, SD) were included as part of daily feed intake. Steers were fed 3 diets during the study; high (starter diet; 63 days), medium (transition diet; 21 days), and low (finisher diet; 63 days) forage TMRs, which are typical life-stage TMRs of growing beef steers (Table 1). Samples from the three diets and alfalfa pellets were collected once per week and bags of *A*. *taxiformis* were randomly sampled and analyzed (Table 2) for dry matter, acid detergent fiber, NDF, lignin, starch, crude fat, total digestible nutrient and mineral content (Cumberland Valley Analytical Services, Waynesboro, PA). Steers were offered water *ad libitum*.

**Table 1:** Ingredients of the experimental diet containing high, medium, and low forage concentrations (% of DM).

Ingredients	High forage	Medium forage	Low forage
*Forage*			
Alfalfa hay	35.0	25.0	5.00
Wheat hay	25.0	15.0	6.00
Dry distiller grain	12.0	14.0	6.00
*Concentrate*			
Rolled corn	20.0	37.0	72.0
Molasses	5.0	5.00	3.00
Fat	1.5	2.00	3.00
Urea	0.35	0.40	1.80
Beef trace salt^1^	0.32	0.32	1.00
Calcium carbonate	0.82	1.15	1.80
Magnesium oxide			0.20
Potassium chloride			0.50

^1^Beef Trace Salt sourced from A.L Gilbert (Oakdale, California) contains; salt manganous oxide, vegetable oil, zinc oxide, copper sulfate, ethylene, diamine dihydriodide, sodium selenite.

**Table 2:** Nutritional composition of experimental diets, *Asparagopsis taxiformis*, and alfalfa pellets.

	High Forage	Medium Forage	Low Forage	Pellets	*A*. *taxiformis*
*% Dry matter*					
Organic matter	92.1	93.1	94.8	88.6	50.9
Crude protein	17.2	17.4	13.2	17.1	16.8
ADF	22.6	16.7	10.5	28.1	11.5
NDF	33.1	25.8	18.6	35.9	33.7
Lignin	4.08	3.05	1.73	6.16	4.08
Starch	16.9	25.0	46.7	0.90	0.35
Crude fat	4.92	6.04	6.77	3.02	0.63
Calcium	0.77	1.00	0.50	2.06	5.29
Phosphorus	0.33	0.38	0.28	0.24	0.18
Magnesium	0.38	0.38	0.23	0.37	0.81
Potassium	1.74	1.42	0.94	2.10	2.02
Sodium	0.18	0.25	0.30	0.20	6.34
*Parts per million*					
Iron	438	335	127	1508	8494
Manganese	61.7	56.0	64.0	88.0	142.5
Zinc	43.2	51.50	58.0	19.0	53.5
Copper	8.67	8.00	7.00	10.0	22.5

Chemical analysis was performed by Cumberland Valley Analytical Services, Waynesboro, PA.

The *A*. *taxiformis* used as a feed additive was provided by Commonwealth Scientific and Industrial Research Organization (CSIRO) Australia. The seaweed was collected during the gametophyte phase from Humpy Island, Keppel Bay, QLD (23o13’01"S, 150o54’01"E) by Center for Macroalgal Resources and Biotechnology of James Cook University, Townsville, Queensland, Australia. Once collected, the *A*. *taxiformis* was frozen, stored at −15°C, then freeze dried at Forager Food Co., Red Hills, Tasmania, Australia, and later ground using a Hobart D340 mixer (Troy, OH, USA) and 3mm sieve. Total seaweed inclusion ranged from 46.7 to 55.7 g/day for Low and 76.1 to 99.4 g/day for High treatment. The seaweed used in the study contained bromoform at a concentration of 7.8 mg/g dry weight as determined by Bigelow Analytical Services (East Boothbay, ME, USA). To increase palatability and adhesion to feed, 200 ml of molasses and 200 ml of water was mixed with the *A*. *taxiformis* supplement, then the molasses-water-*A*. *taxiformis* mixture was homogenously incorporated into the TMR, by hand mixing, for each treatment animal. The Control group also received 200 ml of both molasses and water with their daily feed to ensure *A*. *taxiformis* was the only difference between the three treatments.

### Sample collection and analysis

Methane, CO_2_, and H_2_ gas emissions from steers were measured using the GreenFeed system (C-Lock Inc., Rapid City, SD, USA). Gas emissions were measured during the covariate (baseline) period and experimental period during weeks 3, 6, 9, 12, 15, 18, and 21. In each measurement period, gas emission data were collected during 3 consecutive days as follows: starting at 0700, 1300, and 1900 hours (sampling day 1); 0100, 1000, and 1600 hours (sampling day 2); and 2200 and 0400 hours (sampling day 3). Eructated gas samples from each steer were taken at random across each treatment group. The GreenFeed machine was manually moved to each steer pen where the steer was allowed to enter the machine by choice and induced to eat from the machine for 3 to 5 minutes, followed by a 2-minute background gas sample collection. One GreenFeed unit was used for all gas emissions samples and took approximately 140 minutes to complete each timepoint. The GreenFeed system was calibrated before each measurement period with a standard gas mixture containing (mol %): 5000 ppm CO_2_, 500 ppm CH_4_, 10 ppm H_2_, 21% O_2_ and nitrogen as a balance (Air Liquide America Specialty Gases, Rancho Cucamonga, CA). Recovery rates for CO_2_, CH_4_, and H_2_ observed in this study were within +/− 3% of the known quantities of gas released. Alfalfa pellets were offered at each sampling event as bait feed and was kept below 10% of the total DMI during each 3-day measurement period. The composition of alfalfa pellets is shown in Table 2. Feed residuals were collected daily before the morning feeding to determine the previous day’s intake. Feed intake and feed costs were recorded daily and bodyweight (BW) was measured once weekly, using a Silencer Ranch Model hydraulic squeeze chute (Dubas Equipment Stapleton, NE) equipped with a scale, at 0500 before morning feeding to reduce variability due to gut fill.

After the feeding trial was completed, all 20 steers were sent to a USDA-inspected commercial packing plant (Cargill Meat Solutions, Fresno, California) for slaughter. On the day of slaughter, steers were marked and followed throughout the process. On the first day, livers were collected, placed in individually labelled freezer bags and stored on dry ice until placed in a −20°C freezer. Carcasses were aged for 48 hours in a large cooler and then graded by a certified USDA grader. Directly after grading, carcasses were sent to fabrication where the strip loin from the left side of each carcass was cut and saved for further analysis. All 20 strip loins were vacuum packed then stored on ice and transported back to the University of California, Davis where they were cryovac packaged and stored at 4°C in dark for 14 days. After 14-day of aging, strip loins were cut into steaks (2.45 cm thickness) and individually vacuum packaged and stored at −20°C. Samples of steaks and livers were analyzed by Bigelow Analytical Services (East Boothbay, ME, USA) for bromoform concentrations following a modified protocol described by Paul et al. (2009) [25]. The limits of bromoform detection and quantification were 0.06 mg/kg and 0.20 mg/kg, respectively. Steaks were also analyzed for proximate analysis by Midwest Labs (Omeha, Nebraska) for moisture (AOAC 950.46), protein (AOAC 992.15), fat (AOAC 991.36), ash (AOAC 900.02, 920.155, 920.153), calories (21 CFR P101.9), carbohydrates (100 –Moisture–Protein–Fat—Ash), and iodine (USP 233) concentration.

To test for objective tenderness, slice shear force (SSF) and Warner-Brazler shear force (WBSF) were measured following the protocol described by [34]. One steak from each animal was thawed overnight and cooked to an internal temperature of 71°C. Within 1 to 2 minutes after cooking, the SSF were measured using machine texture analyzer (TMS Pro Texture Analyzer, Food Technology corporation, Sterling, VA, USA) with a crosshead at the speed of 500 mm/minute. To test WBSF, cooked steaks were cooled at 4°C overnight, and then four cores were cut using WEN 8-inch 5 Speed Drill Press from one steak from each animal parallel to the muscle fiber orientation. The WBSF was measured using the TMS Pro texture analyzer with a Warner Bratzler blade (2.8 mm wide) and a crosshead at speed of 250 mm/minute. The average peak forces for all four cores were recorded.

A consumer sensory panel was conducted at UC-Davis. Strip steaks were thawed at 4°C for 24 hours then cooked to an internal temperature of 71°C using a George Foreman clamshell (Spectrum Brands, Middleton, WS, USA). Internal temperature was taken from the geometric center of each steak using a K thermocouple thermometer (AccuTuff 351, model 35100, Cooper-Atkins Corporation, Middlefield, CT, USA). Following cooking, steaks were rested for 3 minutes then cut into 1.5 cm^3^ pieces. Each steak was randomly assigned a unique three digit number, placed into glass bowls covered in tin foil then stored in an insulated food warmer (Carlisle model PC300N03, Oklahoma, OK, USA) for longer than 30 minutes prior to the start of each sensory evaluation session. A total of 112 participants evaluated steak samples during one of the 5 sessions held over a 4-day period. Each participant evaluated a total of three steak samples, one from each treatment group, with a minimum of two 1.5 cm^3^ pieces per steak. Each participant was asked to evaluate tenderness, flavor, juiciness, and overall acceptance using a 9-point hedonic scale (1 = Dislike extremely and 9 = Like extremely).

### Statistical analysis

Statistical analysis was performed using R statistical software (version 3.6.1; The R Foundation for Statistical Computing, Vienna, Austria). The linear mixed-effects models (lme) procedure was used with the steer as the experimental unit. GreenFeed emission data were averaged per steer and gas measurement period, which was then used in the statistical analysis. The statistical model included treatment, diet, treatment × diet interactions, and the covariate term, with the error term assumed to be normally distributed with mean = 0 and constant variance. Individual animal was used as random effect, whereas all other factors were considered fixed. Data was analyzed as repeated measures with an autoregressive 1 correlation structure. Statistical significance was established when *P* ≤ 0.05 and a trend at 0.05 > *P* ≤ 0.10. The consumer sensory evaluation data were analyzed using the Kruskal-Wallis test. The Dunn’s test with *P*-value adjustment following Bonferroni methods was used for post-hoc pair-wise comparisons.

Dry matter intake (DMI) and cost per kg of gain (CPG) data was averaged by week and used in the statistical analysis. Average daily gain (ADG) was calculated by subtracting initial BW from final BW then dividing by the number of experimental days for each diet regimen and the duration of the study (i.e. 63 days on high forage (starter) TMR, 21 days on medium forage (transition) TMR, then 63 days on low forage (finisher) TMR with total study duration of 147 days). Feed conversion efficiency (FCE) was calculated by dividing ADG by DMI for each diet regimen and the duration of the study. Carbon Dioxide (CO_2_), CH_4_, and H_2_ emissions are reported as production (g/day), yield (g/kg DMI), and intensity (g/kg ADG).

## Results

### Gas parameters

**Fig 2:**
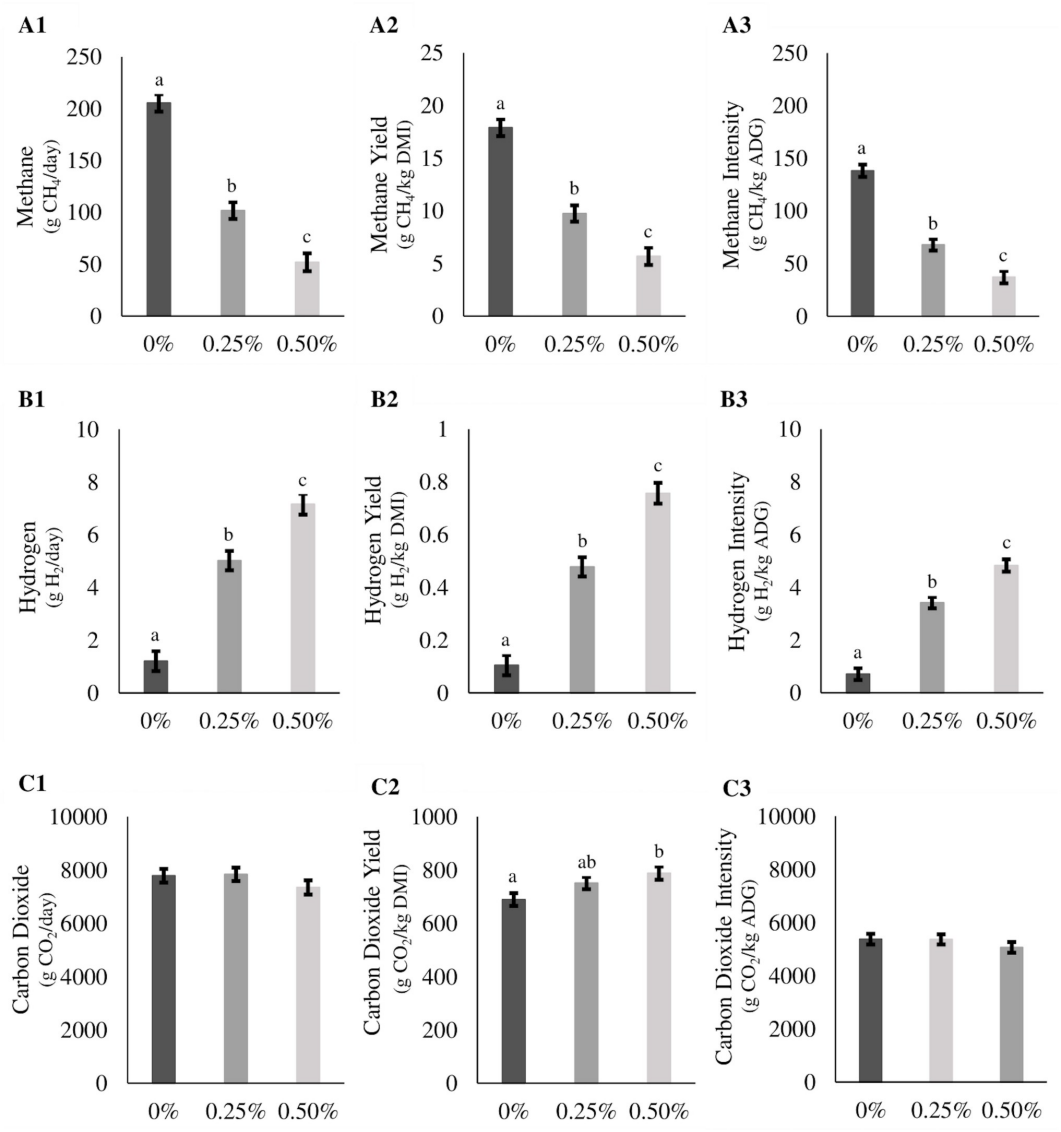
*Asparagopsis taxiformis* inclusion effects on methane, hydrogen and carbon dioxide emissions over a 147-day period. Means, standard deviations, and statistical differences of methane, hydrogen, and carbon dioxide production (g/d) (A1,B1,C1), yield (g/kg dry matter intake (DMI)) (A2,B2,C2), and intensity (g/kg average daily gain) (A3,B3,C3) for 0%, 0.25% (Low), and 0.50% (High) *Asparagopsis taxiformis* inclusion. Means within a graph with different alphabets differ (*P* < 0.05).

The emissions as production (g/day), yield (g/kg DMI), and intensity (g/kg ADG) of CH_4_, H_2_, and CO_2_ gases from the steers in the three treatment groups (Control, Low, and High) are presented in Fig 2 (for the duration of the trial) and Table 3 (divided by the three diet regimes). Inclusion of *A*. *taxiformis* in the TMR had a significant linear reduction in enteric CH_4_ production, yield, and intensity. For the duration of the experimental period, CH_4_ production, yield and intensity declined by 50.6 and 74.9%, 45 and 68%, and 50.9 and 73.1% for Low and High treatments, respectively, compared to Control. Hydrogen production, yield, and intensity significantly increased by 318 and 497%, 336 and 590%, and 380 and 578% in the Low and High treatments, respectively, for the duration of the experiment. Carbon dioxide (CO_2_) production and intensity factors were not affected by either Low or High treatments, however, CO_2_ yield was significantly greater in High treatment compared to Control (*P* = 0.03).

**Table 3:** Effect of *Asparagopsis taxiformis* inclusion at 0.25% (Low) and 0.5% (High) feed organic matter on enteric gas emissions using high-, medium-, and low- forage diets.

*Gas Emission Data*	High Forage					Medium Forage					Low Forage				
	Control^†^	Low^†^	High^‡^	SEM*	P**	Control^†^	Low^†^	High^‡^	SEM*	P**	Control^†^	Low^†^	High^‡^	SEM*	P**
Methane															
Production (g/day)	237^a^	151^b^	98.0^c^	11.4	<0.01	241^a^	116^b^	31.9^c^	15.3	<0.01	139^a^	38.4^b^	25.2^b^	11.4	<0.01
Yield (g/kg DMI)	22.1^a^	14.9^b^	10.6^c^	1.02	<0.01	19.2^a^	10.6^b^	3.92^c^	1.36	<0.01	12.4^a^	3.75^b^	2.50^b^	1.02	<0.01
Intensity (g/kg ADG)	150^a^	94.6^b^	65.3^c^	7.92	<0.01	176^a^	80.3^b^	31.0^c^	12.4	<0.01	88.6^a^	28.8^b^	15.4^b^	7.93	<0.01
Hydrogen															
Production (g/day)	1.25^a^	3.48^b^	5.77^c^	0.44	0.01	1.38^a^	5.88^b^	8.76^c^	0.56	<0.01	0.97^a^	5.71^b^	6.94^b^	0.44	<0.01
Yield (g/kg DMI)	0.12^a^	0.35^b^	0.68^c^	0.05	0.02	0.11^a^	0.55^b^	0.93^c^	0.06	<0.01	0.09^a^	0.53^b^	0.66^b^	0.05	<0.01
Intensity (g/kg ADG)	0.60^a^	2.15^b^	3.77^c^	0.29	0.01	0.93^a^	4.11^b^	6.77^c^	0.42	<0.01	0.60^a^	4.00^b^	3.96^b^	0.29	<0.01
Carbon dioxide															
Production (g/day)	7422	7399	7035	324	1.00	8393	8335	7185	365	0.67	7577	7770	7795	324	1.00
Yield (g/kg DMI)	706	742	815	27.3	0.57	694	779	806	32.9	0.43	678	731	744	27.3	0.78
Intensity (g/kg ADG)	4884	4658	4689	272	1.00	6460	5932	5874	442	1.00	4784	5507	4532	272	0.62

^†^ Control and Low; n = 7 steers per treatment;

^‡^High; n = 6 steers.

*Standard Error of the mean; standard error pooled across treatments.

**P values are pooled across treatments.

^a,b,^^c^ superscripts = note significant differences (P = <0.05) between treatment groups.

An interaction was observed between diet formulation and magnitude of CH_4_ reduction and H_2_ formation for production, yield, and intensity factors (Table 3). Methane production, yield, and intensity in steers on the high forage TMR and supplemented with *A*. *taxiformis* reduced by 36.4 and 58.7%, 32.7 and 51.9%, and 36.9 and 56.4% for Low and High treatments, respectively. Hydrogen production, yield, and intensity increased by 177 and 360%, 198 and 478%, and 256 and 524% for the Low and High treatments, respectively. Methane production, yield, and intensity in steers fed the medium forage TMR and supplemented with *A*. *taxiformis* was reduced by 51.8 and 86.8%, 44.6 and 79.7%, and 54.4% and 82.4%% for the Low and High treatments, respectively. Furthermore, H_2_ production, yield and intensity significantly increased by 326 and 535%, 404 and 753%, and 341 and 626% for the Low and High treatments, respectively. Steers fed low forage TMR and supplemented with *A*. *taxiformis* reduced CH_4_ production, yield, and intensity by 72.4 and 81.9%, 69.8 and 80.0%, and 67.5 and 82.6% for Low and High treatments, respectively. Additionally, H_2_ production, yield, and intensity increased by 419 and 618%, 503 and 649%, and 566 and 559% for the Low and High treatments, respectively. No significant differences were found in CO_2_ production, yield, or intensity in any of the three diets.

### Animal production parameters

Dry matter intake (DMI), ADG, feed conversion efficiency (ADG/DMI; FCE) and cost per gain ($USD/kg weight gain; CPG) as impacted by treatment groups (Control, Low, and High) for the entire experimental period is presented in Table 4 and for the individual TMRs in Table 5. Initial BW, final BW, carcass weight and total weight gained are shown in Table 4. During the entire experiment (Table 4), DMI in Low treatment tended (*P* = 0.08) to decrease by 8% and High treatment DMI significantly reduced by 14% (*P* < 0.01) whereas no significant effects were observed in ADG by either Low or High treatment groups when compared to Control. With the reduction of DMI in Low and High treatments and similar ADG among all 3 treatments, FCE tended to increase 7% (*P* = 0.06) in Low treatment and increased 14% (*P* < 0.01) in High treatment. No significant differences between initial BW, final BW, total gains, CPG or carcass weight were found between Control and treatment groups. While no significant differences were found in CPG, there was a $0.37 USD/kg gain differential between High and Control and $0.18 USD/kg gain differential between Low and Control.

**Table 4:** Effect of *Asparagopsis taxiformis* inclusion at 0.25% (Low), and 0.5% (High) feed organic matter on beef animal parameters over 21 weeks.

*Animal Parameters*	Control^†^	Low^†^	High^‡^	SEM*	P**
Initial BW (kg)	357	348	350	9.21	0.78
Final BW (kg)	589	572	587	11.1	0.73
Total gain (kg)	232	224	236	6.09	0.72
ADG (kg/day)	1.6	1.52	1.56	0.06	0.72
DMI (kg/day)	11.3^a^	10.4^ab^	9.69^b^	0.29	0.04
FCE (ADG/DMI)	0.14^a^	0.15^a^	0.16^b^	0.01	0.04
CPG ($/kg gain)	2.25	2.07	1.88	0.18	0.53
Carcass weight (kg)	370	361	350	13.4	0.61

BW, body weight; ADG, average daily gain; DMI, dry matter intake; FCE, feed conversion efficiency; CPG, cost per gain.

^†^ Control and Low; n = 7 steers per treatment;

^‡^High; n = 6 steers.

*Standard Error of the mean; standard error pooled across treatments.

**P values are pooled across treatments.

^a,b,c^ superscripts = note significant differences (P = <0.05) between treatment groups.

**Table 5:** Effect of *Asparagopsis taxiformis* inclusion at 0.25% (Low) and 0.5% (High) feed organic matter on beef animal parameters using high, medium-, and low- forage diets.

*Animal Parameters*^1^	High Forage					Medium Forage					Low Forage				
	Control^†^	Low^†^	High^‡^	SEM*	P**	Control^†^	Low^†^	High^‡^	SEM*	P**	Control^†^	Low^†^	High^‡^	SEM*	P**
DMI (kg/day)	10.3^a^	9.69^ab^	8.40^b^	0.33	0.34	12.2^a^	10.8^ab^	9.99^b^	0.33	0.05	11.5^a^	10.8^ab^	10.7^b^	0.33	0.34
ADG (kg/day)	1.58	1.61	1.53	0.11	1.00	1.62	1.50	1.38	0.11	0.94	1.60	1.44	1.75	0.11	0.82
FCE (ADG/DMI)	0.15	0.17	0.18	0.01	0.78	0.13	0.14	0.14	0.01	1.00	0.14	0.13	0.17	0.01	0.63
CPG ($/kg ADG)	1.83	1.68	1.54	0.25	0.97	2.67	2.18	2.20	0.26	1.00	2.24	2.35	1.90	0.27	0.95

^1^DMI, dry matter intake; ADG, average daily gain, FCE, feed conversion efficiency; CPG, cost per gain.

^†^ Control and Low; n = 7 steers per treatment;

^‡^High; n = 6 steers.

*Standard Error of the mean; standard error pooled across treatments.

**P values are pooled across treatments.

^a,b,c^ superscripts = note significant differences (P = <0.05) between treatment groups.

Decreases in DMI were also found over the three different TMR diets (Table 5) where steers fed the high and medium forage TMR and the High treatment decreased their DMI 18.5 (*P* = 0.01) and 18.0% (*P* < 0.01), respectively. No significant effects were observed in ADG, CPG, or FCE by the Low or High treatment groups during the individual TMR diets. Additionally, cost differentials for High treatment were $0.29, $0.40, and $0.34 USD/kg gain and for Low treatment were $0.15, $0.49, and $0.34 USD/kg gain for the high, medium, and low forage TMRs, respectively.

### Carcass and meat quality parameters

There was no statistical difference between treatment groups for rib eye area (Table 6). No effects were found between Control, Low, and High treatments in moisture, protein, fat, ash, carbohydrates, or calorie content of strip loins (Table 6). The average WBSF values for the Control, Low and High groups were 2.81, 2.66 and 2.61 kg, respectively. Additionally, the SSF averages were measured as 17.1 for Control, 16.75 for Low and 17.4 kg for High treatments. No significant differences (*P* > 0.05) were found in shear force resistance among treatment groups. Mean scores of all sensory attributes (tenderness, juiciness, and flavor) by consumer panels were not significantly different (*P* > 0.05) among treatment groups (Table 6). The taste panel considered all steaks, regardless of treatment group, to be moderately tender and juicy. This was consistent with the taste panel stating that they moderately liked the flavor of all steaks regardless of treatment group. There was no difference (*P* > 0.05) in overall acceptability among treatment groups. There was a linear increase in iodine concentrations in both Low (*P* < 0.01) and High (*P* < 0.01) compared to Control. Iodine concentrations for the Control treatment group were below detection levels, which was set at 0.10 mg/g (Table 6). However, 5 out of 7 steers in Low treatment group had iodine levels above the detection level with a treatment average of 0.08 mg/g (*P* < 0.01). All 6 steers in the High treatment group were found to contain iodine levels above the detection level with concentration levels ranging between 0.14–0.17 mg/g with a mean of 0.15 mg/g (*P* < 0.01). Bromoform concentrations for all treatment groups were below detection levels, which were 0.06 mg/kg.

**Table 6:** Effect of *Asparagopsis taxiformis* inclusion at 0.25% (Low) and 0.5% (High) on carcass quality, proximate analysis, shear force, and consumer panel preference.

*Carcass Quality*	Control^†^	Low^†^	High^‡^	SEM*	P**
Rib eye area (cm)	28.7	28.4	26.9	0.37	0.65
*Proximate Analysis*					
Moisture (g/100g)	53.9	55.4	55.3	1.70	0.86
Protein (g/100g)	16.1	17.1	17.2	0.70	0.68
Fat (g/100g)	29.1	26.1	26.3	2.20	0.73
Ash (g/100g)	0.73	0.86	0.88	0.05	0.92
Carbohydrates (g/100g)	0.24	1.01	0.42	0.38	0.57
Calories	327	307	307	17.7	0.79
Iodine (PPM)	0^a^	0.08^b^	0.15^c^	0.02	<0.01
*Shear Force*					
Warner-Bratzler (kgf)	2.81	2.66	2.61	0.24	0.82
Slice Shear Force (kgf)	17.1	16.8	17.4	1.87	0.98
*Consumer Panel* ^1^					
Tenderness	6.72	6.68	6.45	0.17	0.37
Juiciness	6.35	6.33	6.07	0.17	0.40
Flavor	6.63	6.34	6.24	0.15	0.10
Overall	6.66	6.36	6.46	0.16	0.26

^†^ Control and Low; n = 7 steers per treatment;

^‡^High; n = 6 steers.

*Standard Error of the mean; standard error pooled across treatments.

**P values are pooled across treatments.

^a,b,c^ superscripts = note significant differences (P = <0.05) between treatment groups.

^1^ A 9-point hedonic scale was used (1 = Dislike extremely and 9 = like extremely).

## Discussion

### Enteric methane production, yield, and intensity

**Fig 3:**
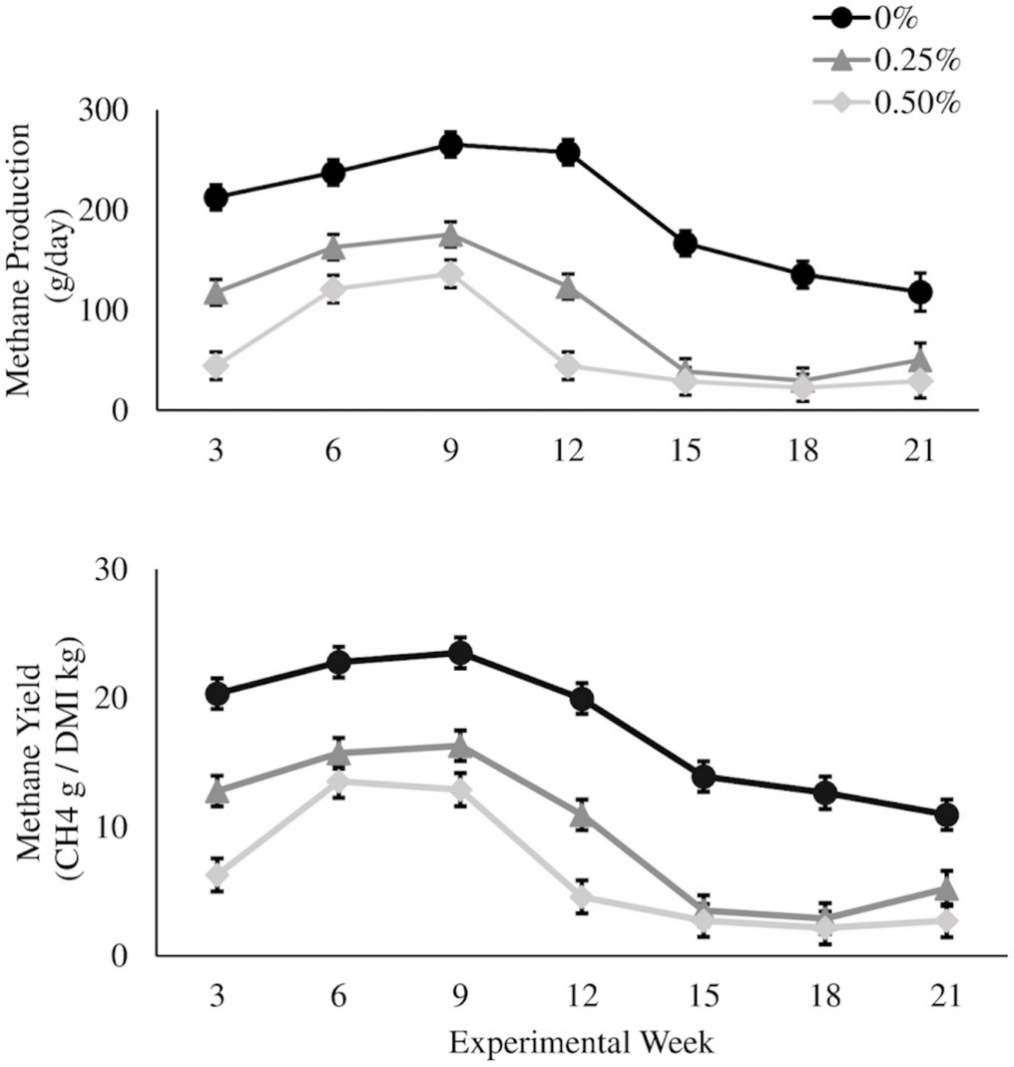
*Asparagopsis taxiformis* inclusion effects on methane emissions during the 21 week experimental period. Methane production [g CH_4_/day] (A) and methane yield [g CH_4_/kg DMI] (B) from beef steers supplemented with *Asparagopsis taxiformis* at 0%, 0.25%, and 0.5% of basal total mixed ration on an organic matter basis during the 21 week experimental period. Data points are treatment means for each gas collection timepoint and error bars represent standard errors.

This study demonstrated that dietary inclusion of *A*. *taxiformis* induces a consistent and considerable reduction in enteric CH_4_ production from steers on a typical feedlot style diet. Enteric CH_4_ is the largest contributor to GHG emissions from livestock production systems. Significant reductions in CH_4_ yield, which is standardized by DMI, when *Asparagopsis* is supplemented to beef cattle diets has been established in this study and are similar to the reductions found in previous studies [6,29,35]. While CH_4_ intensities have been previously reported for dairy cows fed *A*. *armata* [29], this is the first study to measure CH_4_ intensity differences in beef cattle fed *A*. *taxiformis*. Intensity reports are important to determine the amount of methane being produced per unit of output for ruminant livestock systems. There is a concern that feed additives and other CH_4_ reducing agents decrease in efficacy over time [14]. This study provided evidence that the seaweed inclusion was effective in reducing CH_4_ emissions, which persisted for the duration of the study of 147 days (Fig 3). Notably, until this study the longest exposure to *A*. *taxiformis* had been demonstrated for steers in a study ending after a 90-d finishing period [6]. To date, only three *in vivo* studies have been published using *Asparagopsis* spp. to reduce enteric CH_4_ emissions in feedlot Brangus steers [6], lactating dairy cattle [29], and sheep [35]. All studies show considerable yet variable reductions in enteric CH_4_ emissions. The differences in efficacy are likely due to levels of seaweed inclusion, formulation of the diets, and differences in seaweed quality based on bromoform concentrations.

It has been previously hypothesized that NDF levels can also influence the rate at which CH_4_ is reduced with the inclusion of inhibitors [4,24]. In the current study, the magnitude of reductions in CH_4_ production were negatively correlated (r^2^ = 0.89) with NDF levels in the 3 diet regimens that contained 33.1% (high forage), 25.8% (medium forage), and 18.6% (low forage) NDF levels. Enteric CH_4_ production was reduced 32.7, 44.6 and 69.8% in steers on the Low treatment and 51.9, 79.7, and 80.0% on High treatment with high, medium and low forage TMRs, respectively. The low forage TMR, containing the lowest NDF levels, was the most sensitive to the inclusion of *A*. *taxiformis* with CH_4_ reductions above 70% at equivalent inclusion levels compared to the higher forage TMRs. Vyas et al (2018) showed similar trends of greater methane reduction potential in high grain, low NDF, diets in combination with the anti-methanogenic compound 3-NOP [24]. It has been hypothesized to increase efficacy by a reduction in rumen MCR concentration when low NDF is fed, thus increasing the MCR targeting capability of the anti-methanogenic feed additive. An 80.6% reduction of CH_4_ yield in sheep fed diets containing 55.6% NDF, however, the level of *A*. *taxiformis* intake by the sheep was unclear but was offered at 6 times the High treatment in our study [35]. A 42.7% reduction in CH_4_ yield was observed in lactating dairy cattle fed a diet containing 30.1% NDF at 1% inclusion rate of *A*. *armata* [29]. The high forage TMR in our study had a similar NDF level to the dairy study, however, had approximately double the reduction of CH_4_, even when consuming 50% less seaweed. These differences relate to a large degree to the quality of seaweed in terms of the concentration of bromoform, which was 1.32 mg/g in the dairy study [29] compared to 7.82 mg/g in the current study. The same collection of *A*. *taxiformis* was used in a previously published *in vivo* study focused on Brangus feedlot steers for a duration of 90 days [6]. This seaweed had bromoform concentration of 6.55 mg/g, which was marginally lower than our study and may be due to variation in the collection, sampling, analysis techniques, or storage conditions. Despite the marginally lower bromoform concentration in the seaweed and using 0.20% inclusion rate of *A*. *taxiformis* on OM basis, the CH_4_ yield was reduced by up to 98% in Brangus feedlot steers. The diet used by Kinley et al. (2020) included 30.6% NDF, which was similar to our high fiber diet [6]. The greater efficacy of *A*. *taxiformis* in that study could be due to collective feed formulation differences such as the energy dense component of barley versus corn, which is typical of Australian and American feedlots, respectively. Additionally, it could be due to beneficial interaction with the ionophore, monensin, that was used in the Australian study. Monensin has not been used in any other feed formulation in other *in vivo* studies with the inclusion of *Asparagopsis* species. Use of monensin in diets has shown to decrease CH_4_ yields by up to 6% in feedlot steers while also having an enhanced effect in diets containing greater NDF levels [36]. A potential enhancing interaction of the seaweed with monensin is of great interest and further investigation will elucidate this potential that could have significant beneficial economic and environmental impact for formulated feeding systems that use monensin.

### Enteric hydrogen and carbon dioxide emissions

**Fig 4:**
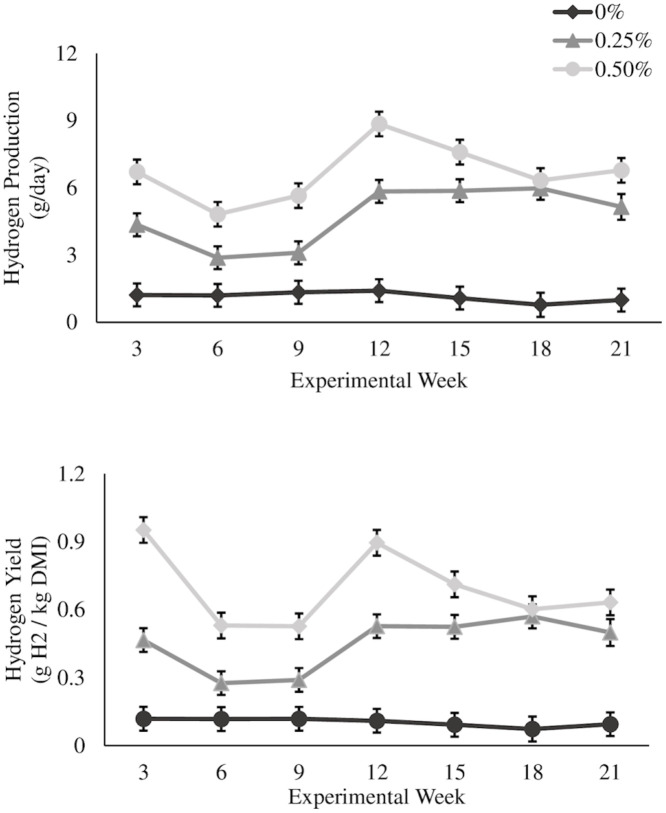
*Asparagopsis taxiformis* inclusion effects on hydrogen emissions during the 21-week experimental period. Hydrogen production [g H_2_/day] (A) and Hydrogen yield [g H_2_/kg DMI] (B) from beef steers supplemented with *Asparagopsis taxiformis* at 0%, 0.25%, and 0.5% of basal total mixed ration on an organic matter basis during the 21 week experimental period. Data points are treatment means for each gas collection timepoint and error bars represent standard errors.

Increases in H_2_ yield have typically been recorded when anti-methanogenic feed additives are used, and with the addition of *Asparagopsis* species in dairy cattle (1.25–3.75 fold) [29] and Brangus feedlot steers (3.8–17.0 fold) [6]. Similar increases in H_2_ yield have been reported in feed additives that reduce enteric CH_4_ emissions targeting methanogens. For example, in lactating dairy cows supplemented with 3-NOP, H_2_ yield increased 23–71 fold [37]. Bromochloromethane (BCM) fed to goats increased H_2_ (mmol/head per day) 5–35 fold, while chloroform fed to Brahman steers increased H_2_ yield 316 fold [38,39]. Although feeding *Asparagopsis spp*. increased overall H_2_ yield (Fig 4), the magnitude was considerably lower (1.25–17 fold) compared to alternative CH_4_ reducing feed additives (5–316 fold), with similar levels of reductions in CH_4_. This indicates that there may be a redirection of H_2_ molecules that would otherwise be utilized through the formation of CH_4_ and redirected into different pathways that could be beneficial to the animal. For example, increased propionate to acetate concentrations have been recorded in *in vitro* [40,41] and *in vivo* [6] using *A*. *taxiformis* and BCM [42] for CH_4_ mitigation which may indicate that some of the excess H_2_ is being utilized for propionate production.

Similar to the lactating dairy cattle study with 1% *A*. *armata* supplementation [29], the CO_2_ yield in the current study also increased in the High group (Fig 2). However, in the current study no differences in CO_2_ production were seen. Typically, CO_2_ and H_2_ are used in the methanogenesis pathway to form CH_4_ thus increases in exhaled CO_2_ is expected with the addition of anti-methanogenic compounds. The fact that only CO_2_ yield increased may be due to decreases in DMI, which could have reduced overall CO_2_ generation thus resulting in no increases seen in CO_2_ production factors.

### Animal production parameters

Dry matter intake reductions observed in this study were consistent with previous studies in lactating dairy cows where decreases in DMI were found to be 10.7 and 37.9% at 0.50 and 1.0% inclusion rate of *A*. *armata* [29], respectively. Decreases in DMI have also been reported in cattle fed other anti-methanogenic feed additives in a linear dose-response fashion. For example, Tomkins et al (2009) reported 3 to 19% reductions in DMI in steers supplemented with BCM at dosages between 0.15 and 0.60 g/100 kg live weight [5]. Additionally, Martinez-Fernandez et al (2016) found 1.7 to 15% reductions in DMI when chloroform was directly applied to the rumen, through a rumen fistula, at dosages between 1 to 2.6 g/100g liveweight [39]. In contrast, Kinley et al (2020) reported no significant differences in DMI at the highest *A*. *taxiformis* level of 0.20% [6]. However, the inclusion level was less than our study’s lowest inclusion rate, so based on previous experiment’s observation of reduced DMI in a dose-response manner [29], it was expected to have lower effect on DMI. Decreases in DMI are normally associated with lower productivity due to lower levels of nutrients and dietary energy consumed. However, there was no significant difference in ADG between steers in the High treatment and Control (average 1.56 kg/day) groups despite consuming 14% less feed. The results were in agreement with a previous study [29], in which milk production was not compromised at a 0.5% OM inclusion level despite reductions in DMI. The FCE (ADG/DMI) increased significantly in High treatment group, suggesting that inclusion rates of *A*. *taxiformis* at 0.5% improves overall feed efficiency in growing beef steers. Since a large proportion of on farm costs is the purchase of feed, an improved feed efficiency is particularly exciting for producers to decrease feed costs while also producing the same amount of total weight gains. Total gains were between 224 kg (Low) to 236 kg (High) combined with an average cost differential of ~$0.18 USD/kg gain (Low) and ~$0.37 USD/kg gain (High). A producer finishing 1000 head of beef cattle has the potential to reduce feed costs by $40,320 (Low) to $87,320 (High) depending on seaweed dosage. While the CPG in this study were not statistically significant, this may be due to low animal numbers in each treatment and warrants further investigation on a larger feedlot setting to reduce animal variability.

### Bromoform and iodine residues

Bromoform is the major active ingredient responsible for CH_4_ reduction when fed to cattle [43]. However, high levels of bromoform are suspected to be hazardous for humans and mice. While bromoform intake limits are yet to be defined for cattle specifically, the US EPA (2017) has suggested a reference dose for bromoform, an estimated level of daily oral exposure without negative effects, to be 0.02 mg/kg BW/day for human consumption [44]. It is essential that food products from livestock consuming the seaweed are confirmed as safe for consumption and that bromoform residues are not transferred to the edible tissues and offal of bovines at levels detrimental to food safety. Previous studies have demonstrated that bromoform was not detectable in the kidney, muscle, fat deposits, blood, feces, and milk in either Brangus feedlot steers [6], dairy cows [29], or sheep [35]. Strip loin and liver samples from steers were collected and in agreement with previous studies, no bromoform was detected in this study.

The National Academies of Sciences, Engineering, and Medicine recommendations for daily iodine intake in growing beef cattle is 0.5 mg/g DMI and maximum tolerable limit is 50 mg/g DMI [45]. Based on DMI intake from steers in this study, recommended daily iodine intake levels were 5.2 mg/day and 4.85 mg/day and maximum limits are 521 mg/day and 485 mg/day for Low and High treatment groups, respectively. The iodine level in the *A*. *taxiformis* fed in the current study contained 2.27 mg/g, therefore, maximum daily intake of seaweed iodine was 106–127 mg/day and 173–225 mg/day for the Low and High treatment groups, respectively. While these levels do not exceed maximum tolerable limits, they exceed daily iodine intake recommendations for cattle, therefore it was appropriate to test for iodine residue levels in meat used for human consumption. The US Food and Nutrition Board of the National Academy of Sciences has set a tolerable upper intake level (UL) for human consumption of foods, which is defined as the highest level of daily intake that poses no adverse health effects [46]. The iodine UL ranges between 200 ug/day to 1,100 ug/day depending on age, gender, and lactation demographics. Strip loins tested for iodine residues had levels of 0.08 and 0.15 ug/g from steers in treatments Low and High, respectively. These iodine residues are far under the UL limits for human consumption. For example, UL for a person under 3 years of age is 200 ug/day meaning that this person would have to consume more than 2.5 kg/day and 1.3 kg/day of meat from a Low and High steers, respectively, to reach the UL. An adult over the age of 18 has an UL of 1,100 ug/day and would have to consume more than 13.8 kg/day and 7.3 kg/day of meat from a Low and High steers, respectively, to reach their UL of iodine intake. At the inclusion levels and iodine concentration of *A*. *taxiformis* used in this study the margin of safety is extremely high and the likelihood of iodine toxicity from consuming the meat is extremely low. The health hazards of consistently consuming any meat at such levels is much higher than the iodine toxicity risks. Low level iodine in meat may provide for provision of iodine to populations that suffer from natural iodine deficiency, a common issue in populations with low intake of marine food products [47].

### Carcass and meat quality parameters

Marbling scores ranged from 410–810 while all carcasses, regardless of treatment, graded as either choice or prime. The value placed on tenderness in the marketplace is high and has even been found that consumers are likely to pay premiums for more tender beef [48]. Many factors can greatly affect meat tenderness, such as animals’ age at slaughter, breed, marbling, and diet [49–51]. All animals used in the current study were of similar age and breed. Additionally, no significant difference in average marbling scores was observed. The lack of significant differences seen in these factors further supports that the supplementation of *A*. *taxiformis* at the current dosage did not impact the tenderness of meat. This is in agreement with Kinley et al. (2020)’s meat taste assessment where no differences between Control and *A*. *taxiformis* supplemented beef cattle were found [6]. The combination of both the current study as well as the Kinley et al (2020) study [6] indicates that the supplementation of *A*. *taxiformis* at or below 0.5% to cattle does not significantly impact overall meat quality nor alter the sensory properties of the steaks.

## Conclusions

This study demonstrated that the use of *A*. *taxiformis* supplemented to beef cattle diets reduced enteric CH_4_ emissions for a duration of 21 weeks without any loss in efficacy. The efficacy was highly correlated with the proportion of NDF in the diet as demonstrated through the typical stepwise transition to a feedlot finishing diet formulation. Additionally, supplementing *A*. *taxiformis* had no measurable bromoform residues, no detrimental iodine residual effects in the product, and did not alter meat quality or sensory properties. Importantly, the use of *A*. *taxiformis* impacts DMI and not ADG, therefore increasing overall feed efficiency (FCE) in growing beef steers. This study also demonstrated a potential to reduce the cost of production per kg of weight gain. These feed cost reductions in combination with significantly reduced CH_4_ emissions have a potential to transform beef production into a more economically and environmentally sustainable red meat industry.

Next steps for the use of *Asparagopsis* as a feed-additive would be to develop aquaculture techniques in ocean and land-based systems globally, each addressing local challenges to produce a consistent and high-quality product. Processing techniques are evolving with the aim of stabilizing as feed supplement and the economics of the supply chain. The techniques include utilization of already fed components as carriers and formats such as suspensions in oil which may be done using fresh or dried seaweed, and options in typical feed formulations such as mixtures are being explored [52]. Transportation of the processed or unprocessed seaweed should be kept to a minimum, so cultivation in the region of use is recommended specially to avoid long-haul shipping.

## Supporting information

S1 Table Original data. Data sets used for statistical analysis of animal production, gas emissions, carcass parameters, and taste panel between Control, 0.25% OM inclusion of *Asparagopsis taxiformis* (Low), and 0.50% OM inclusion of *Asparagopsis taxiformis* (High) treatment groups.
(XLSX)

## References

[1] Gerber PJ, Steinfeld H, Henderson B, Mottet A, Opio C, Dijkman J, et al. Tackling Climate Change through Livestock: A Global Assessment of Emissions and Mitigation Opportunities. FAO, Rome. 2013.

[2] Moraes LE, Strathe AB, Fadel JG, Casper DP, Kebreab E. Prediction of enteric methane emissions from cattle. Glob. Change Biol. 2014;20(7):2140e2148. 10.1111/gcb.12471 24259373

[3] Hristov AN, Oh J, Firkins JL, Dijkstra J, Kebreab E, Waghorn G, et al. Mitigation of methane and nitrous oxide emissions from animal operations: I. A review of enteric methane mitigation options. J. Anim. Sci. 2013;(91):5045–5069. 10.2527/jas.2013-6583 24045497

[4] Dijkstra J, Bannink A, France J, Kebreab E, van Gastelen S. Short communication: antimethanogenic effects of 3-nitrooxypropanol depend on supplementation dose, dietary fiber content, and cattle type. J. Dairy Sci. 2018;101:9041e9047. 10.3168/jds.2018-14456 30055923

[5] Tomkins NW, Colegate SM, Hunter RA. A bromochloromethane formulation reduces enteric methanogenesis in cattle fed grain-based diets. Anim. Prod. Sci. 2009;49(12):1053–1058. 10.1071/EA08223

[6] Kinley RD, Martinez-Fernandez G, Matthews MK, de Nys R, Magnusson M, Tomkins NW. Mitigating the carbon footprint and improving productivity of ruminant livestock agriculture using a red seaweed. J. Clean. Prod. 2020;(59):120836. 10.1016/j.jclepro.2020.120836

[7] Duin EC, Wagner T, Shima S, Prakash D, Cronin B, Yáñez-Ruiz DR, et al. Mode of action uncovered for the specific reduction of methane emissions from ruminants by the small molecule 3-nitrooxypropanol. Proc. Natl. Acad. Sci. 2016;(113):6172e6177. 10.1073/pnas.1600298113 27140643 PMC4896709

[8] Smith EL, Mervyn L, Johnson AW, Shaw N. Partial synthesis of vitamin B12 coenzyme and analogues. Nature 1962;194(4834);1175–1175. 10.1038/1941175a0 13914206

[9] Wood JM, Kennedy FS, Wolfe RS. Reaction of multihalogenated hydrocarbons with free and bound reduced vitamin B12. Biochemistry. 1968;7(5):1707–1713. 10.1021/bi00845a013 4870333

[10] Johnson ED, Wood AS, Stone JB, Moran ET Jr.. Some effects of methane inhibition in ruminants (steers). Can. J. Anim. Sci. 1972;52(4):703–712. 10.4141/cjas72-083

[11] Ermler U, Grabarse W, Shima S, Goubeaud M, Thauer RK. Crystal structure of methyl-coenzyme M reductase: the key enzyme of biological methane formation. Science. 1997;278(5342):1457e1462. 10.1126/science.278.5342.1457 9367957

[12] Liu H, Wang J, Wang A, Chen J. Chemical inhibitors of methanogenesis and putative applications. Appl. Microbiol. Biotechnol. 2011;(89):1333e1340. 10.1007/s00253-010-3066-5 21193988

[13] Abecia L, Toral PG, Martín-García AI, Martínez G, Tomkins NW, Molina-Alcaide E, et al. Effect of bromochloromethane on methane emission, rumen fermentation pattern, milk yield, and fatty acid profile in lactating dairy goats. J. Dairy Sci. 2012;95(4):2027–2036. 10.3168/jds.2011-4831 22459848

[14] Knight T, Ronimus RS, Dey D, Tootill C, Naylor G, Evans P, et al. Chloroform decreases rumen methanogenesis and methanogen populations without altering rumen function in cattle. Anim. Feed Sci. Technol. 2011;166:101–112. 10.1016/j.anifeedsci.2011.04.059

[15] Russell JB, Wallace RJ. Energy-yielding and energy-consuming reactions: The rumen microbial ecosystem Springer Dordrecht, Chicago 1997. p. 246–282.

[16] Van Soest PJ. Nutritional ecology of the ruminant. Cornell University Press. 1994.

[17] Blaxter KL, Clapperton JL. Prediction of the amount of methane produced by ruminants. Br. J. Nutr. 1965;19(1):511–522. 10.1079/bjn19650046 5852118

[18] Johnson KA, Johnson DE. Methane emissions from cattle. J. Anim. Sci. 1995;73(8):2483–2492. 10.2527/1995.7382483x 8567486

[19] Bannink A, Kogut J, Dijkstra J, France J, Kebreab E, Van Vuuren AM, et al. Estimation of the stoichiometry of volatile fatty acid production in the rumen of lactating cows. J. Theor. Biol. 2006;238(1):6–51. 10.1016/j.jtbi.2005.05.026 16111711

[20] Bannink A, France J, Lopez S, Gerrits WJJ, Kebreab E, Tamminga S, et al. Modelling the implications of feeding strategy on rumen fermentation and functioning of the rumen wall. Anim. Feed Sci. Technol. 2008;143(4):3–26. 10.1016/j.anifeedsci.2007.05.002

[21] Niu M, Kebreab E, Hristov AN, Oh J, Arndt C, Bannink A. Prediction of enteric methane production, yield, and intensity in dairy cattle using an intercontinental database. Glob. Change Biol. 2018 8;24(8):3368–89. 10.1111/gcb.14094 29450980 PMC6055644

[22] Hungate R. Chapter VI—Quantities of Carbohydrate Fermentation Products. In: The Rumen and its Microbes. Acad. Press. 1966. p. 245–280.

[23] Janssen PH. Influence of hydrogen on rumen methane formation and fermentation balances through microbial growth kinetics and fermentation thermodynamics. Anim. Feed Sci. Technol. 2010;(160):1–22. 10.1016/j.anifeedsci.2010.07.002

[24] Vyas D, McGinn SM, Duval SM, Kindermann MK, Beauchemin KA. Optimal dose of 3-nitrooxypropanol for decreasing enteric methane emissions from beef cattle fed high-forage and high-grain diets. Animal Production Science. 2018;58(6):1049–55.

[25] Paul NA, Cole L, de Nys R, Steinberg PD. Ultrastructure of the gland cells of the red alga *Asparagopsis armata* (*Bonnemaisoniaceae*). J. Phycol. 2006;(42):637–645. 10.1111/j.1529-8817.2006.00226.x

[26] Machado L, Magnusson M, Paul NA, de Nys R, Tomkins N. Effects of Marine and Freshwater Macroalgae on In-Vitro Total Gas and Methane Production. PloS One 2014;(9)1. 10.1371/journal.pone.0085289 24465524 PMC3898960

[27] Kinley RD, Vucko MJ, Machado L, Tomkins NW. In vitro evaluation of the antimethanogenic potency and effects on fermentation of individual and combinations of marine macroalgae. Am. J. Plant Sci. 2016;(7):2038e2054. 10.4236/ajps.2016.714184

[28] Kinley RD, de Nys R, Vucko MJ, Machado L, Tomkins NW. The red macroalgae *Asparagopsis taxiformis* is a potent natural antimethanogenic that reduces methane production during in vitro fermentation with rumen fluid. Anim. Prod. Sci. 2016;(56):282e289. 10.1071/AN15576

[29] Roque BM, Salwen JK, Kinley R, Kebreab E. Inclusion of *Asparagopsis armata* in lactating dairy cows’ diet reduces enteric methane emission by over 50 percent. J. Clean. Prod. 2019;(234):132–138. 10.1016/j.jclepro.2019.06.193

[30] Machado L, Tomkins N, Magnusson M, Midgley D, deNyes R, Rosewarne C. In vitro response of rumen microbiota to the antimethanogenic red macroalga *Asparagopsis taxiformis*. Microb. Ecol. 2018;(75):811–818. 10.1007/s00248-017-1086-8 29018917

[31] Lanigan GW. Metabolism of pyrrolizidine alkaloids in the ovine rumen. IV. Effects of chloral hydrate and halogenated methanes on rumen methanogenesis and alkaloid metabolism in fistulated sheep. Aus. J. of Ag. Res. 1972;23(6):1085–91. 10.1071/AR9721085

[32] Ungerfeld EM, Rust SR, Boone DR, Liu Y. Effects of several inhibitors on pure cultures of ruminal methanogens. J. of Applied Microb. 2004;97(3):520–6. 10.1111/j.1365-2672.2004.02330.x 15281932

[33] Killinger KM, Calkins CR, Umberger WJ, Feuz DM, Eskridge KM. Consumer sensory acceptance and value for beef steaks of similar tenderness, but differing in marbling level. J. Anim. Sci. 2004;82(11):3294–3301. 10.2527/2004.82113294x 15542476

[34] AMSA. Research guidelines for cookery, sensory evaluation, and instrumental tenderness measurements of meat. Am. Meat Sci. Assoc. 2016;1–105.

[35] Li X, Norman HC, Kinley RD, Laurence M, Wilmot M, Bender H, et al. *Asparagopsis taxiformis* decreases enteric methane production from sheep. Anim. Prod. Sci. 2018;58(4):681–688. 10.1071/AN15883

[36] Appuhamy JRN, Strathe AB, Jayasundara S, Wagner-Riddle C, Dijkstra J, France J, et al. Anti-methanogenic effects of monensin in dairy and beef cattle: A meta-analysis. J. Dairy Sci. 2013;96(8):5161–5173. 10.3168/jds.2012-5923 23769353

[37] Hristov AN, Oh J, Giallongo F, Frederick TW, Harper MT, Weeks HL, et al. An inhibitor persistently decreased enteric methane emission from dairy cows with no negative effect on milk production. Proc. Natl. Acad. Sci. 2015;112(34):10663–10668. 10.1073/pnas.1504124112 26229078 PMC4553761

[38] Mitsumori M, Shinkai T, Takenaka A, Enishi O, Higuchi K, Kobayashi Y, et al. Responses in digestion, rumen fermentation and microbial populations to inhibition of methane formation by a halogenated methane analogue. Br. J. Nut. 2012;108(3):482–491. 10.1017/S0007114511005794 22059589

[39] Martinez-Fernandez G, Denman SE, Yang CL, Cheung JE, Mitsumori M, Mcsweeney CS. Methane inhibition alters the microbial community, hydrogen flow, and fermentation response in the rumen of cattle. Front. Microbiol. 2016;7(1122). 10.3389/fmicb.2016.01122 27486452 PMC4949212

[40] Machado L, Magnusson M, Paul NA, Kinley R, de Nys R, Tomkins N. Dose-response effects of *Asparagopsis taxiformis* and *Oedogonium sp*. on in-vitro fermentation and methane production. Journal of Applied Phycology. 2016;28:1443–1452. 10.1007/s10811-015-0639-9

[41] Roque BM, Brooke CG, Ladau J, Polley T, Marsh LJ, Najafi N, et al. Effect of the macroalgae *Asparagopsis taxiformis* on methane production and rumen microbiome assemblage. Anim. Microbiome 2019;1(1):3. 10.1186/s42523-019-0004-4 33499933 PMC7803124

[42] Denman SE, Fernandez GM, Shinkai T, Mitsumori M, McSweeney CS. Metagenomic analysis of the rumen microbial community following inhibition of methane formation by a halogenated methane analog. Front. Microbiol. 2015;6(1087). 10.3389/fmicb.2015.01087 26528253 PMC4602129

[43] Machado L, Magnusson M, Paul NA, Kinley R, de Nys R, Tomkins N. Identification of bioactives from the red seaweed Asparagopsis taxiformis that promote antimethanogenic activity in-vitro. *Journal of Applied Phycology*. 2016;28:3117–3126. 10.1007/s10811-016-0830-7

[44] United States Environmental Protection Agency. Integrated Risk Information System (IRIS) on Bromoform. National Center for Environmental Assessment, Washington, D.C. 2017.

[45] NASEM. Nutrient Requirements of Beef Cattle. 9th Rev. Ed. National Academy Press, Washington, DC. 2016.

[46] Trumbo P, Yates AA, Schlicker S, Poos M. Dietary reference intakes: vitamin A, vitamin K, arsenic, boron, chromium, copper, iodine, iron, manganese, molybdenum, nickel, silicon, vanadium, and zinc. J. Acad. Nutr. Diet. 2001;101(3):294. 10.1016/S0002-8223(01)00078-5 11269606

[47] Pearce EN, editor. Iodine deficiency disorders and their elimination. Springer International Publishing; 2017 Jan 20.

[48] Miller MF, Carr MA, Ramsey CB, Crockett KL, Hoover LC. Consumer thresholds for establishing the value of beef tenderness. J. Anim. Sci. 2001;79(12):3062–3068. 10.2527/2001.79123062x 11811460

[49] Corbin C, Miller M, O’Quinn T, Dinh T, Legako J, Garmyn A, et al. Consumer assessment and fatty acid analysis of beef strip steaks of similar tenderness with varying marbling levels. Meat Sci. 2014;1(96):469. 10.1016/j.meatsci.2013.07.100

[50] Warner RD, Greenwood PL, Pethick DW, Ferguson DM. Genetic and environmental effects on meat quality. Meat Sci. 2010;86(1):171–83. 10.1016/j.meatsci.2010.04.042 20561754

[51] Blank CP, Russell J, Lonergan SM, Hansen SL. Influence of feed efficiency classification and growing and finishing diet type on meat tenderness attributes of beef steers. J Anim Sci. 2017 7;95(7):2986–2992. 10.2527/jas.2016.1312 . 28727083

[52] Magnusson M, Vucko MJ, Neoh TL, de Nys R. Using oil immersion to deliver a naturally-derived, stable bromoform product from the red seaweed Asparagopsis taxiformis. Algal Research. 2020 10 1;51:102065. 10.1016/j.algal.2020.102065

